# Supplementary material for: Green Extraction Techniques of Phytochemicals from Hedera helix L. and In Vitro Characterization of the Extracts
Source: Plants (Basel). 2023 Nov 20;12(22):3908. doi: 10.3390/plants12223908 (PMC10675592; doi:10.3390/plants12223908)
Supplement: Supplementary file 1 [file plants-12-03908-s001.zip › Supplementary material_2730070.pdf]

Green Extraction Techniques of Phytochemicals from *Hedera helix* L. and in vitro Characterization of the Extracts

Adina I. Gavrila [1], Christina M. Zalaru [2], Rodica Tatia [3], Ana-Maria Seciu-Grama [3], Cristina L. Negrea [1], Ioan Calinescu [1], Petre Chipurici [1], Adrian Trifan [1] and Ioana Popa [1,*]

[1] Faculty of Chemical Engineering and Biotechnologies, National University of Science and Technology Politehnica Bucharest, 011061, Bucharest, Romania; adinagav@yahoo.com (A.I.G.); negrea_cristina2001@yahoo.com (C.L.N.); ioan.calinescu@gmail.com (I.C.); petre.chipurici@gmail.com (P.C.); adriantrifan2000@yahoo.com (A.T.)

[2] Department of Organic Chemistry, Biochemistry and Catalysis, Faculty of Chemistry, University of Bucharest, 050663, Bucharest, Romania; chmzalaru@gmail.com (C.Z.);

[2] Department of Cellular and Molecular Biology, National Institute of Research and Development for Biological Sciences, 060031, Bucharest, Romania; rodica.tatia@gmail.com (R.T.); ana.seciu@yahoo.com (A-M.S-G.);

* Correspondence: asofiei.ioana@yahoo.com (I.P.)

Table S1. Factor loadings

	PC1	PC2
Total saponins content (TSC)	**0.930**	0.367
Total carbohydrates content (TCC)	**0.967**	-0.113
Total phenolic content (TPC)	**0.962**	-0.162
Antioxidant activity (AA)	**0.971**	-0.079

Table S2. Factor scores

Method	Method Description	PC1	PC2
1	MAE, 50 °C, 80% Ethanol	2.250	0.273
		2.074	0.175
		2.381	0.091
2	UAE, 50 °C, 80% Ethanol	2.952	-0.254
		2.978	-0.425
		3.126	-0.428
3	CHE, 50 °C, 80% Ethanol	1.363	-0.040
		1.246	0.069
		1.481	-0.144
4	MAE, 50 °C, 80% Ethanol	0.864	0.402

		0.621	0.419
		0.404	0.506
5	UAE, 50 °C, 80% Ethanol	0.790	0.073
		0.719	0.015
		0.380	0.152
6	CHE, 40 °C, 80% Ethanol	-1.998	0.788
		-1.815	0.912
		-1.823	0.811
7	MAE, 50 °C, 0% Ethanol	-1.977	-0.439
		-1.953	-0.477
		-1.852	-0.380
8	UAE, 50 °C, 0% Ethanol	-1.672	-0.480
		-1.430	-0.521
		-1.369	-0.632
9	CHE, 50 °C, 0% Ethanol	-2.489	-0.221
		-2.577	-0.056
		-2.674	-0.188

Table S3. Correlation matrix.

**Variables**	**TSC**	**TCC**	**TPC**	**AA**
TSC	**1**			
TCC	0.856*	**1**		
TPC	0.842*	0.919*	**1**	
AA	0.867*	0.928*	0.922*	**1**

* Significant level at $p<0.05$.
